# The participation of insulin-like growth factor-binding protein 3 released by astrocytes in the pathology of Alzheimer’s disease

**DOI:** 10.1186/s13041-015-0174-2

**Published:** 2015-12-04

**Authors:** Kiwamu Watanabe, Kengo Uemura, Megumi Asada, Masato Maesako, Haruhiko Akiyama, Shun Shimohama, Ryosuke Takahashi, Ayae Kinoshita

**Affiliations:** Department of Neurology, Graduate School of Medicine, Kyoto University, Yoshida-Konoe-cho, Sakyo-ku, Kyoto, 606-8501 Japan; School of Human Health Sciences Faculty of Medicine, Kyoto University, Shogoin-Kawahara-cho, Sakyo-ku, Kyoto, 606-8507 Japan; Tokyo Metropolitan Institute of Medical Science, 2-1-6 Kamikitazawa, Setagaya-ku Tokyo, 156-8506 Japan; Department of Neurology, Sapporo Medical University School of Medicine, 16 Minami-1-jyo-Nishi, Chuo-ku Sapporo, 060-8543 Japan

**Keywords:** Insulin-like growth factor –Ι, Insulin-like growth factor binding protein-3, Amyloid–beta, Calcineurin, Astrocyte, Glycogen synthase kinase-3β, Tau

## Abstract

**Background:**

Alzheimer’s disease (AD) is characterized by senile plaques, extracellular deposits composed primarily of amyloid–beta (Aβ), and neurofibrillary tangles, which are abnormal intracellular inclusions containing hyperphosphorylated tau.

The amyloid cascade hypothesis posits that the deposition of Aβ in the brain parenchyma initiates a sequence of events that leads to dementia. However, the molecular process by which the extracellular accumulation of Aβ peptides promotes intracellular pathologic changes in tau filaments remains unclear. To elucidate this process, we presumed that astrocytes might trigger neuronal reactions, leading to tau phosphorylation. In this study, we examined AD pathology from the perspective of the astrocyte-neuron interaction.

**Results:**

A cytokine-array analysis revealed that Aβ stimulates astrocytes to release several chemical mediators that are primarily related to inflammation and cell adhesion. Among those mediators, insulin-like growth factor (IGF)-binding protein 3 (IGFBP-3) was highly upregulated.

In AD brains, the expression of IGFBP-3 was found to be increased by western blot analysis, and increased expression of IGFBP-3 was observed in astrocytes via fluorescence microscopy.

In addition, we reproduced the increase in IGFBP-3 after treatment with Aβ using human astrocytoma cell lines and found that IGFBP-3 was expressed via calcineurin. In AD brains, the activated forms of calcineurin were found to be increased by western blot analysis, and increased expression of calcineurin was observed in astrocytes via fluorescence microscopy.

When Ser9 of glycogen synthase kinase-3β (GSK-3β) is phosphorylated, GSK-3β is controlled and tau phosphorylation is suppressed. Aβ suppresses the phosphorylation of GSK-3β, leading to tau phosphorylation. In this study, we found that IGF-Ι suppressed tau phosphorylation induced by Aβ, although IGFBP-3 inhibited this property of IGF-Ι. As a result, IGFBP-3 contributed to tau phosphorylation and cell death induced by Aβ.

**Conclusions:**

Our study suggested that calcineurin in astrocytes was activated by Aβ, leading to IGFBP-3 release. We further demonstrated that IGFBP-3 produced by astrocytes induced tau phosphorylation in neurons. Our study provides novel insights into the role of astrocytes in the induction of tau phosphorylation and suggests that IGFBP-3 could be an important link between Aβ and tau pathology and an important therapeutic target.

**Electronic supplementary material:**

The online version of this article (doi:10.1186/s13041-015-0174-2) contains supplementary material, which is available to authorized users.

## Background

Alzheimer’s disease (AD) is a progressive neurodegenerative disease and the most prevalent type of dementia in the elderly population. AD is clinically characterized by cognitive decline and pathologically characterized by senile plaques, neurofibrillary tangles (NFT) and glial cell activation [1–7].

Senile plaques are extracellular deposits that are composed primarily of amyloid–beta (Aβ) [3], which is proteolytically cleaved from amyloid precursor protein (APP) [8, 9]. Aβ is widely considered to be the primary pathological agent in AD [3, 5, 10] because mutations of the genes that encode APP and presenilin, which are enzymes related to the cleavage of Aβ, are known to cause early-onset familial AD [11, 12]. In addition, transgenic mice that overexpress mutated forms of human APP are widely used as AD models.

NFT are abnormal intracellular inclusions that contain hyperphosphorylated tau, which is a microtubule-associated protein [13, 14]. Aβ oligomers are known to be associated with tau hyperphosphorylation [4], which leads to the disruption of the neuronal cytoskeleton, followed by neurodegeneration and cell death [15, 16].

The amyloid cascade hypothesis posits that the deposition of the Aβ peptide in the brain parenchyma initiates a sequence of events that ultimately leads to dementia [17]. However, the molecular process by which the extracellular accumulation of Aβ peptides promotes intracellular pathologic changes in tau filaments remains unknown, although many researchers attempted to elucidate this process. To date, various hypotheses have been proposed to elucidate this mechanism. For instance, Gamblin et al. reported that Aβ peptides promoted pathological tau filament assembly in neurons by triggering caspase-mediated cleavage of tau and generating a proteolytic product with enhanced polymerization kinetics [18]. On the other hand, the astrocyte-neuron interaction has also attracted attention. Astrocytes were reported to be necessary for and to accelerate Aβ-induced tau phosphorylation [19]. Talantova et al. revealed that Aβ engaged α7 nicotinic acetylcholine receptors to induce the release of astrocytic glutamate, which then activated extrasynaptic NMDA receptors on neurons. The activity of these NMDA receptors might reflect early synaptic injury [20]. These studies led us to consider the possibility that environmental factors surrounding neurons might be important for tau phosphorylation. Thus, we presumed that glial cells might trigger neuronal reactions, leading to tau phosphorylation.

Astrocyte activation is inevitably seen in the AD brain [21–26]. Reactive astrocytes are found around amyloid plaques in AD brains, as well as in animal models [27, 28], suggesting that Aβ drives astrocytes to become activated. Aβ can alter glial homeostasis, including the dysregulation of Ca^2+^ signaling [29–31]. One of the key players in Ca^2+^ signaling is calcineurin (CaN), which is a　calcium-related phosphatase downstream of Ca^2+^ [32, 33]. In the literature, CaN was suggested to be involved in AD. Many activated astrocytes in the AD brain and in the mouse AD model express high levels of CaN [34–36]. CaN is known to be elevated during the pathological process of AD [36–39], but several mechanisms have been suggested to underlie this observation. Some reports indicate that Aβ itself may activate CaN signaling in astrocytes [36, 40–43]. In turn, CaN may strongly activate transcription factors involved in cytokine production and phenotype switching, according to other reports [44, 45].

Because astrocytes and microglia are known to produce several proinflammatory signaling molecules [46–48], we used differential antibody analysis to compare the expression levels of these proteins between Aβ42-treated and control peptide-treated astrocytes. One of the highly upregulated proteins in Aβ42-treated astrocytes was insulin-like growth factor (IGF)-binding protein 3 (IGFBP-3), which is known to be involved in IGF signaling. We presume that IGFBP-3, which is released from astrocytes after Aβ42 stimulation, may be a key molecule in the association between astrocytes and neurons in AD. In this study, we demonstrated the following:

1. Activated astrocyte releases IGFBP-3 in response to Aβ via the CaN cascade.
2. IGFBP-3 is localized within astrocytes in the proximity of Aβ plaques and within Aβ plaques themselves in the AD brain.
3. IGFBP-3 induces tau phosphorylation in neurons, which leads to neuronal death.

Thus, we conclude that IGFBP-3 is a key molecule that can link Aβ with NFT and may be a desirable therapeutic target.

## Results

### Aβ stimulates murine astrocytes to release several chemical mediators

Aβ has been proposed to stimulate astrocytes to release several chemical mediators. To identify the chemical mediators released by murine astrocytes after Aβ treatment, we performed a cytokine-array analysis. The culture medium from murine primary astrocytes was collected after a 48-h treatment with 1 μM Aβ_1–42_ or synthetic peptide Aβ_42-1_ (inverted sequence of Aβ) and subjected to analysis.

**Fig. 1:**
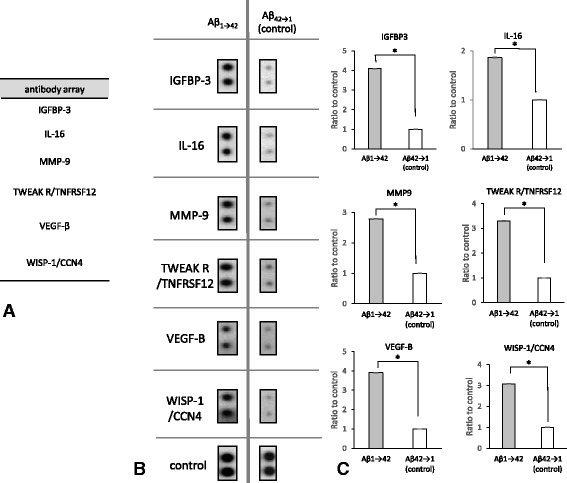
Protein list upregulated by Aβ_1-42_ and their quantification results from a cytokine-array analysis (**a**). The list of reproducibly upregulated proteins was obtained from three independent cytokine-array analyses. Proteins related to inflammation and cell adhesion were shown to be upregulated. Real data and quantitative analyses are shown (**b**, **c**). The culture medium from primary murine astrocytes was collected after a 48-h treatment with 1 μM Aβ_1–42_ or synthetic peptide Aβ_42-1_ (inverted sequence of Aβ) and was subjected to analysis. The left spots are the results obtained for Aβ_1–42_, and the right spots are the results obtained for the control (**b**). On the membrane of the cytokine-array analysis, Anti-HRP and anti-streptavidin are laid out as positive control signals, which can be used to identify the orientation and help normalize the results from the different arrays being compared. As a result of densitometry for these normalized spots, proteins that had significantly increased were selected

Representative data obtained from three independent experiments are shown in Fig. 1b. Quantification data of three experiments are Fig. 1c. Array analyses indicated that the mediators released from astrocytes in response to Aβ_1–42_ were primarily related to inflammation and cell adhesion (Fig. 1a). Among these proteins, IGFBP-3 was highly upregulated and selected for further investigation.

### The expression of IGFBP-3 is increased in AD brains, as indicated by western blotting analysis

**Fig. 2:**
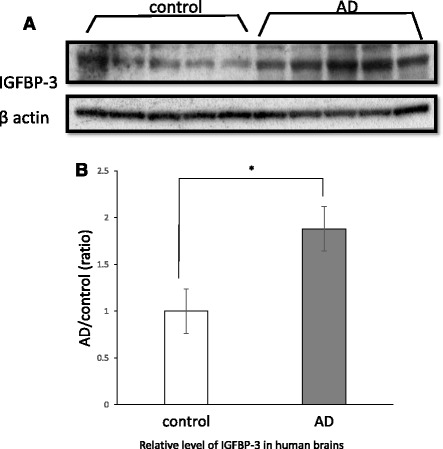
IGFBP-3 expression in human brains. Brain homogenates of temporal cortexes from AD patients (*AD*, *n = 5*) and age-matched non-AD controls (*control*, *n = 5*) were analyzed via western blot using anti-IGFBP3 and β-actin antibodies (**a**). The band densities were quantified using *Image J*. The IGFBP3*/*β-actin ratio was calculated and analyzed using Mann-Whitney’s *U* test. The IGFBP3/β-actin ratio was significantly increased in the brains of AD patients (**b**, 188.1 ± 23.9 %, *p* < 0.05). The data represent the mean ± SE. * indicates statistically significant differences (*p < 0.05)

IGF-binding proteins (IGFBPs) have a strong affinity for IGFs and are known to regulate the bioactivity of IGFs. Serum IGFBP-3, which is one of six IGFBPs, was previously reported to be upregulated in AD patients [49]. To test whether the levels of IGFBP-3 were increased in the AD brain, we analyzed the protein levels of IGFBP-3 in the temporal cortices of human AD patients and age-matched non-AD controls. As expected, western blot analysis revealed increased expression levels of IGFBP-3 in AD brains in comparison to non-AD controls (Fig. 2a). A quantitative analysis demonstrated that the ratio of IGFBP-3 to β-actin was significantly increased in AD brains in comparison to non-AD controls (*n* = 5, 188.1 ± 23.9 %, *p* < 0.05; Fig. 2b).

### Upregulated IGFBP-3 co-localizes with astrocytes in AD patients, as indicated by fluorescence microscopy

**Fig. 3:**
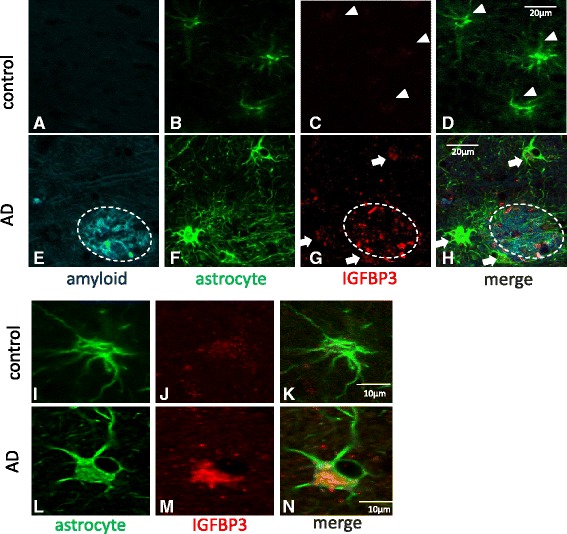
IGFBP-3 in astrocytes from human brains. Confocal images from a triple-fluorescence immunohistochemical study of β-amyloid stained with FSB (blue) (**a** and **e**), astrocytes stained with GFAP (green) (**b**, **f**, **i** and **l**) and IGFBP3 (red) (**c**, **g**, **j** and **m**) are shown. D, H, K and N are merged images. Representative images from a control brain (**a**-**d**) and an AD brain (**e**-**h**) are shown. Higher magnification images from another control (**i**-**k**) and from AD patients (**l**-**n**) are shown. Little IGFBP-3 was detectable in astrocytes from control brains (**c**, **d**, **j** and **k**, white arrowheads), whereas increased expression of IGFBP-3 was observed in astrocytes from AD patients (**g** and **h**, white arrows), especially in gemistocytic astrocytes (**m** and **n**) in the proximity of Aβ plaques. Moreover, IGFBP-3 frequently co-existed with β-amyloid within Aβ plaques (**g** and **h**, white circle). These human brain samples were from the temporal cortex

Next, we asked whether IGFBP-3 expression was actually upregulated in astrocytes from AD brains using fluorescence microscopy. Triple-labeling experiments (IGFBP-3, GFAP, and Aβ) revealed that fewer astrocytes were immunolabeled with anti-IGFBP-3 in control brains (Fig. 3). In contrast, increased expression of IGFBP-3 was observed in astrocytes from AD patients, especially in gemistocytic astrocytes in the proximity of Aβ plaques. Moreover, IGFBP-3 was frequently observed within Aβ plaques (Fig. 3). These results indicate that IGFBP-3 is upregulated in astrocytes from AD brains.

### Aβ stimulates human astrocytoma cell lines to upregulate IGFBP-3 at both the mRNA and protein levels

**Fig. 4:**
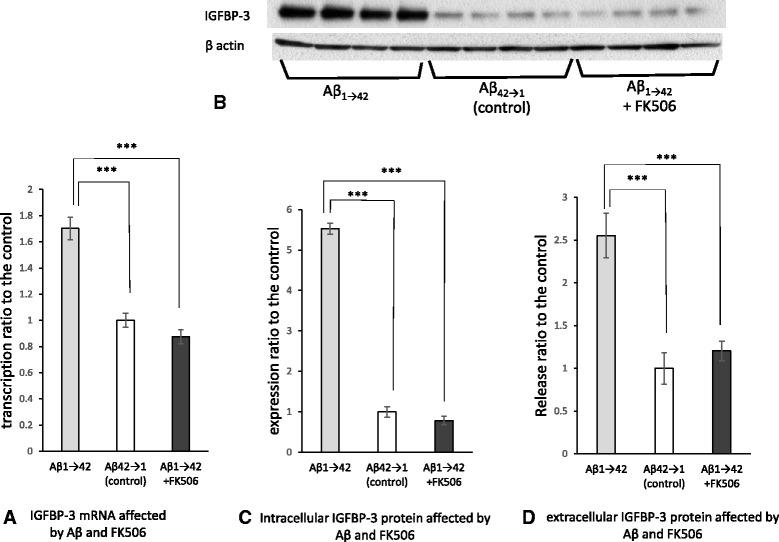
mRNA of intra- and extracellular IGFBP-3 are increased by Aβ via calcineurin in cultured cells. Human astrocytoma cells (H4 cells) were treated with 1 μM Aβ_1–42_ or control peptide for 24 h. mRNA was extracted and subjected to quantitative RT-PCR. The levels of IGFBP-3 mRNA were significantly increased after treatment with Aβ_1–42_, compared with the control peptide (**a**, *n* = 6, 170.2 ± 8.7 %, *p* < 0.001). Simultaneous treatment with both 1 μM Aβ_1–42_ and 1 μM FK506 for 24 h downregulated the levels of IGFBP-3 mRNA, compared with Aβ_1–42_ treatment alone (**a**, *n* = 6, *p* < 0.001). The data represent the mean ± SE. * indicates statistically significant differences (***p < 0.001). **b**-**d** Western blotting and ELISA were performed to verify the upregulation of both intra- and extracellular IGFBP-3 protein levels after 48 h of treatment with 1 μM Aβ_1–42_ or control peptide. Increased levels of IGFBP-3 were observed in the Aβ_1–42_-treated cell preparations in comparison to preparations treated with the control peptide treatment, as indicated by western blot (**c**, *n* = 4, 552.5 ± 14.1 %, *p* < 0.001) and ELISA (**d**, *n* = 7, 255.4 ± 26.0 %, *p* < 0.001). FK506 treatment significantly reduced the levels of intra- and extracellular IGFBP-3, as demonstrated by western blot (**c**, *n* = 4, *p* < 0.001) and ELISA (**d**, n = 7, *p* < 0.001), respectively. The data represent the mean ± SE. * indicates statistically significant differences (***p < 0.001)

To determine whether Aβ treatment upregulates the transcription of IGFBP-3, we treated human astrocytoma cells (H4 cells) with 1 μM Aβ_1–42_ for 24 h. After treatment, mRNA was obtained from the cells, and the samples were subsequently subjected to quantitative RT-PCR analysis. Fig. 4a shows that the levels of IGFBP-3 mRNA were significantly increased after treatment with Aβ_1–42_, as compared with a control peptide (*n* = 6, 170.2 ± 8.7 %, *p* < 0.001 in comparison to the control). To verify IGFBP-3 upregulation at the protein level, we treated H4 cells with 1 μM Aβ_1–42_ for 48 h and subjected the cell lysate and the medium to western blot and ELISA analyses, respectively. We observed increased levels of IGFBP-3 in the Aβ_1–42_-treated cell preparations in comparison to control peptide- treated preparations using western blot (*n* = 4, 552.5 ± 14.1 %, *p* < 0.001) and ELISA (*n* = 7, 255.4 ± 26.0 %, *p* < 0.001) (Fig. 4b, c and d).

### FK506 treatment of human astrocytoma cells attenuates the effect of Aβ on IGFBP-3 at both the mRNA and the protein levels

Previous reports showed that CaN levels increased during the time course of AD [36–39]. CaN is also known to be highly expressed in many activated astrocytes in the AD brain and in the mouse model of AD [34–36]. Thus, we focused on the CaN cascade as one of the pathways through which astrocytes release IGFBP-3 in response to Aβ treatment. We examined whether the CaN inhibitor FK506 could affect the release of IGFBP-3 from astrocytes. We treated H4 cells with 1 μM Aβ_1–42_ and 1 μM FK506 for 24 h. Real-time PCR revealed that the levels of IGFBP-3 mRNA were significantly reduced after treatment with FK506, in comparison to cells that were not treated with FK506 (*n* = 6, *p* < 0.001; Fig. 4a). To examine the effect of FK506 on intracellular and extracellular expression of the IGFBP-3 protein, we treated H4 cells with 1 μM Aβ_1–42_ and FK506 for 48 h. The levels of intra- and extra-cellular IGFBP-3 were found to be reduced in FK506-treated cells in comparison to untreated cells, as indicated by western blot (*n* = 4, *p* < 0.001) and ELISA (*n* = 7, *p* < 0.001), respectively (Fig. 4b, c and d). These data suggested that astrocytes expressed IGFBP-3 via the CaN cascade.

### Activated forms of CaN are increased in AD brains

**Fig. 5:**
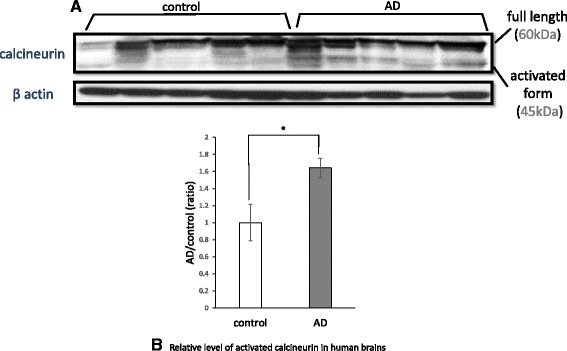
Calcineurin activation in human brains. Brain homogenates of the temporal cortex from AD patients (*AD*, *n = 5*) and age-matched non-AD controls (*control*, *n = 5*) were analyzed via western blot using anti-calcineurin and anti-β-actin antibodies (**a**). The 45 kDa bands represent the activated form of calcineurin, and the 60 kDa bands represent full-length calcineurin. The band densities of calcineurin were quantified using *Image J*. The ratio of the activated form to full-length calcineurin (*activated form/full length*) was calculated and analyzed using Mann-Whitney’s *U* test. The activated form/full length ratio was significantly increased in the brains of AD patients (**b**, 164.1 ± 11.4 %, *p* < 0.05). The data represent the mean ± SE. * indicates statistically significant differences (*p < 0.05)

To confirm that the CaN cascade is actually activated in the AD brain, we analyzed the levels of activated CaN in temporal cortices from human AD patients in comparison to samples from age-matched non-AD controls. CaN is composed of a 60-kDa catalytic subunit (PPP3CA) and a 19 kDa calcium-binding regulatory subunit (PPP3R1) [50]. The 60-kDa catalytic subunit possesses a catalytic domain, a regulatory subunit binding domain, a calmodulin binding domain, and an autoinhibitory domain at the C-terminus [51]. When Ca^2+^ and calmodulin bind to CaN, the autoinhibitory domain is detached and CaN becomes activated. Activated CaN is identified as a 45 kDa protein by western blot. Fig. 5a shows that the activated forms of CaN were increased in AD brains in comparison to non-AD controls (*n* = 5, 164.1 ± 11.4 %, *p* < 0.05; Fig. 5b).

### Upregulated CaN co-localizes with astrocytes in AD patients, as indicated by fluorescence microscopy

**Fig. 6:**
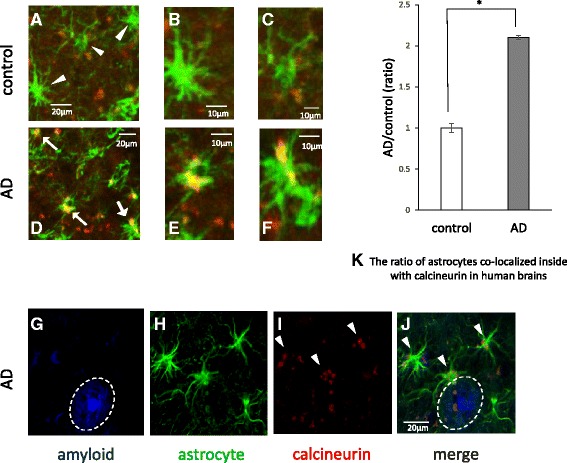
Calcineurin in astrocytes from human brains. Confocal merged images of the double-fluorescence immunohistochemical study of GFAP (green) and calcineurin (CaN) (red). Representative images from a control brain (**a**-**c**) and an AD brain (**d**-**f**) are shown. B, C, E and F are higher magnification images. Little CaN was found in astrocytes from control brains (**a**-**c**), whereas increased expression of CaN was observed in astrocytes from AD patients (**d**-**f**) Triple-fluorescence labeling of β-amyloid with FSB (blue) (**g**), GFAP (green) (**h**) and CaN (red) (**i**) in AD brains revealed the upregulation of CaN in astrocytes (I and J, white arrow) in the proximity of Aβ plaques (**g** and **j**, white circle). We took nine independent randomly chosen low magnification images from each of four patients and counted the number of astrocytes that expressed CaN out of the total number of astrocytes. More astrocytes express CaN in AD brains than in control brains (*n* = 5, 210.3 ± 2.2 %, *p* < 0.05). The data represent the mean ± SE. * indicates statistically significant differences (*p < 0.05). These human brain samples were from the temporal cortex

Next, we examined whether CaN expression was actually upregulated in astrocytes from AD brains using fluorescence microscopy. As expected, increased expression of CaN was localized to astrocytes from AD patients, especially in proximity to Aβ plaques, although a small amount of CaN co-localized with astrocytes from control brains (Fig. 6a-6). We counted the number of GFAP-positive cells that expressed CaN and compared this value between AD and control samples. More astrocytes expressed CaN in AD brains than in control brains (*n* = 5, 210.3 ± 2.2 %, *p* < 0.05; Fig. 6k).

### IGF-Ι suppresses glycogen synthase kinase-3β and tau phosphorylation, although IGFBP-3 counteracts the effect of IGF-Ι

IGF signaling is known to be closely linked to glycogen synthase kinase-3β (GSK-3β) signaling. IGF-I stimulation was reported to induce prolonged phosphorylation of GSK-3β (at Ser^9^) via the PI3K pathway [52]. GSK-3β is a well-known phosphorylase of the tau protein. Previous reports have shown that IGF-Ι inhibits GSK-3β in neural cells, resulting in tau hyperphosphorylation [53, 54]. Given that IGFBP-3 acts as an inhibitor of IGF signaling, we focused on GSK-3β signaling to examine the effect of IGFBP-3 on tau phosphorylation in primary neurons.

**Fig. 7:**
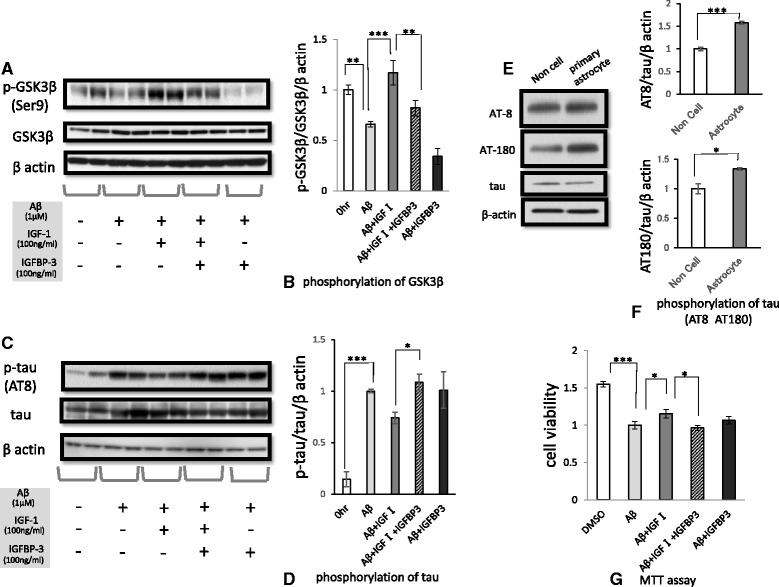
The effects of IGFBP-3 on the phosphorylation of GSK-3β and tau, and on cell death. Primary murine neurons were treated with Aβ_1–42_ (1 μM)_,_ IGF-1 (100 ng/ml) and IGFBP-3 (100 ng/ml), as designated in the figure, for 48 h. Western blotting analysis of primary murine neurons revealed that Aβ reduced the inhibitory phosphorylation of GSK-3β at Ser9 (n = 6, p < 0.01), but IGF-1 restored the level of Ser9 phosphorylation (**a** and **b**, *p* < 0.001). The ratio of phosphorylated GSK3β to the total, normalized by β actin, is shown, converting the value of pre-treatment into 1(**b**). IGFBP-3 precluded IGF-induced Ser9 phosphorylation (*p* < 0.01). On the other hand, Aβ induced tau phosphorylation of murine primary neurons after 48 h of treatment (**c** and **d**, *n* = 6, *p* < 0.001). The ratio of phosphorylated tau to the total, normalized by β actin, is shown, converting the value of pre-treatment into 1(**d**). Although IGF-1 suppressed tau phosphorylation, IGFBP-3 counteracted the effect of IGF-1 (**c** and **d**, *p* < 0.05).To examine phosphorylation of tau in murine primary neuron when treated by astrocyte-cultured media, two dishes were prepared, one on which primary astrocytes spread and the other on which no cell spread. The culture medium was each collected after a 48-h treatment with 1 μM Aβ. Primary neurons were treated with the media which added 100 ng/ml IGF-Ι for 48 h and subjected the cell lysate to western blot. Astrocyte-cultured media induced tau phosphorylation in primary murine neurons in comparison to the control.as detected by both the AT8 antibody and AT180 antibody (**e**). The ratio to the control of AT8 (n = 3 157.34 ± 3.44 %, *p* < 0.001) and AT180 (n = 3 133.63 ± 1.96 %, *p* < 0.05), respectively, are shown, which value was corrected by total tau and βactin (**f**). Primary murine neurons were treated with Aβ_1–42_, IGF-1 and IGFBP-3 for 72 h and subsequently were subjected to an MTT assay to examine cell death (*n* = 16). IGF-1 attenuated the neuronal cell death induced by Aβ (155.1 ± 5.7 %, *p* < 0.05), but IGFBP-3 inhibited the pro-survival effect of IGF-1 (**g**, *p* < 0.05). The data represented by the mean ± SE. * indicates statistically significant differences (*p < 0.05, **p < 0.01, ***p < 0.001)

GSK-3β activity is known to be controlled by inhibitory phosphorylation at the Ser9 residue. We examined the effect of IGF-Ι and/or IGFBP-3 on inhibitory phosphorylation of GSK-3β. Interestingly, Aβ reduced Ser9 phosphorylation of GSK-3β (*n* = 6, p < 0.01), but IGF-Ι restored the levels of Ser9 phosphorylation (*p* < 0.001; Fig. 7a and b,). IGFBP-3 precluded the IGF-induced Ser9 phosphorylation (*p* < 0.01).

**Fig. 8:**
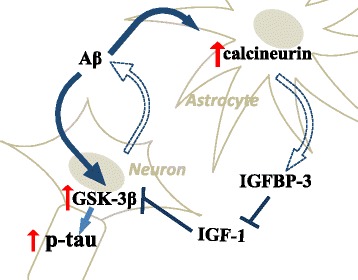
Hypothetical model of AD pathogenesis from the perspective of the astrocyte-neuron interaction. The hypothetical pathway by which Aβ affects both astrocytes and neurons, thereby activating GSK3β and leading to tau phosphorylation. Aβ, which is released from neurons, may induce astrocytes to release IGFBP-3 by activating calcineurin. Aβ may also directly activate GSK-3β, resulting in tau phosphorylation. IGF-1 can suppress GSK-3β and prevent tau phosphorylation, whereas IGFBP-3 impedes the protective effect of IGF-1

Because the hyperphosphorylation of tau is characteristic of AD pathology, we examined tau phosphorylation. As reported previously, Aβ induced tau phosphorylation in primary murine neurons after 48 h of treatment, as detected by the AT8 antibody (Fig. 7c). We further investigated the effect of IGF-Ι and/or IGFBP-3 on Aβ-induced phosphorylation of tau. Although IGF-Ι tended to suppress tau phosphorylation, IGFBP-3 counteracted the effect of IGF-Ι (*p* < 0.05; Fig. 7c and d). We did the same experiment by using another antibody, AT180, which identified different phosphorylation site of tau from AT8. The results are shown in supplementary figure (Additional file 1). This result was compatible with the one from AT8. Because both phosphorylation sites are controlled by GSK-3β, this result supports that IGF-Ι and IGFBP-3 affect GSK-3β, which leads to tau phosphorylation.

### Astrocyte-cultured media induce tau phosphorylation in primary murine neurons

In AD brain, IGFBP-3 released from astrocytes is supposed to actually induce phosphorylation of tau in neurons. To reproduce this reaction, we did an additional experiment in vitro. We examined phosphorylation of tau in murine primary neuron when treated by astrocyte-cultured media. Given regarded non cell-cultured media as control, we found astrocyte-cultured media induced tau phosphorylation in primary murine neurons in comparison to the control.as detected by both the AT8 antibody and AT180 antibody (Fig. 7e).

Figure 7F are the ratio to the control of AT8 (*n* = 3 157.34 ± 3.44 %, *p* < 0.001) and AT180 (*n* = 3 133.63 ± 1.96 %, *p* < 0.05), respectively, which value was corrected by total tau and βactin. This result is compatible with the one for IGFBP-3 (Fig. 7c and d).

### IGFBP-3 inhibits the pro-survival effect of IGF-Ι

Next, we performed an MTT assay to examine the influence of IGFBP-3 on cell death. IGF-Ι rescued primary murine neurons from Aβ–induced cell death (*n* = 16, *p* < 0.05). On the other hand, IGFBP-3 inhibited the pro-survival effect of IGF-Ι (*p* < 0.05; Fig. 7g). This result was compatible with the tau phosphorylation results.

## Discussion

An accumulating number of reports suggest that Aβ is involved in AD pathogenesis. Aβ, observed in the brain parenchyma, is generally believed to initiate a sequence of events that leads to dementia and a sequence of pathological changes including NFT. However, the molecular process that directly connects Aβ and NFT has not yet been revealed. We thought that the key player must exist, who acted as a bridge between extracellular occurrences and intracellular changes. We then focused on astrocytes to investigate the link between Aβ and tau pathology from the perspective of the interrelationship between neurons and astrocytes. Garwood et al. suggested that it was possible that astrocyte-derived cytokines bound to neuronal plasma membrane receptors, inducing specific signaling pathways that resulted in the activation of tau kinases implicated in aberrant tau phosphorylation [19]. We propose a novel pathway in which released Aβ induces IGFBP-3 in astrocytes, which leads to tau phosphorylation via GSK-3β (Fig. 8).

Reactive astrocytes are frequently found around amyloid plaques in both AD brains and animal models [27, 28], suggesting that Aβ may activate astrocytes. Aβ was reported to cause the deregulation of cellular Ca^2+^ signaling [29–31]. One of the Ca^2+^-dependent protein phosphatases is CaN [32, 33]. CaN is activated by Ca^2+^ and calmodulin binding. Liu et al. reported that CaN A underwent proteolysis by activated calpain I at lysine 501 to generate a 57-kDa truncated form in the AD brain, and this cleavage markedly activated the Ca^2+^/calmodulin-dependent phosphatase activity of CaN [37]. In this study, we also confirmed that activated forms of CaN were increased in AD brains in comparison to non-AD controls (Fig. 5). Moreover, we determined that the upregulated CaN was localized in astrocytes from AD patients, especially in proximity to Aβ plaques; in contrast, little CaN co-localized with astrocytes in control brains (Fig. 6). CaN strongly activates transcription factors involved in cytokine production, phenotype switching, and many other processes [44, 45]. In the case of the overexpression of active CaN, most astrocyte-related growth factors/cytokines or their receptors are upregulated, suggesting that CaN acts upstream of these factors [34]. It is emphasized that neuroinflammation significantly contributes to AD pathogenesis and cytokines and chemical mediators play a key role in inflammatory processes in AD [46–48]. This time, we identified the chemical mediators released by murine astrocytes after Aβ treatment. Among them, we focused on IGFBP-3 and examined whether or not IGFBP-3 was the downstream of CaN. By inhibiting CaN, the levels of both IGFBP-3 mRNA and protein were significantly downregulated (Fig. 4), indicating that astrocytes expressed IGFBP-3 via the CaN cascade.

The IGFs are growth-promoting peptides that share significant structural homology with insulin. Unlike insulin, IGFs circulating in the plasma bind IGFBPs to form the IGF-IGFBP complex [55]. IGFBPs have a specifically high affinity for IGFs and are known to regulate the bioactivity of IGFs both positively and negatively [56–60]. IGFBP-3 is most abundant among the six different subtypes of IGFBPs [61, 62].

IGF-I promotes neuronal survival largely through the phosphoinositide 3-kinase (PI3K)/AKT signaling pathway [58]. The binding of IGF-I to its receptor, IGF-IR, activates AKT. Activated AKT phosphorylates and inhibits GSK-3β. GSK-3β mediates the hyper-phosphorylation of tau [63] and impairs learning and memory by preventing the induction of long-term potentiation [64]. The IGF system is neuroprotective against Aβ-induced cell death [65] and beneficial for cognition [66].

Conversely, attenuation of IGF signaling is presumed to lead to neurotoxic. Given insulin resistance is defined as a condition in which insulin signals fail to work, despite the presence of a sufficient amount of insulin.[67], reduced cellular responsiveness to IGF is put into IGF resistance. Insulin resistance has been reported to be implicated in the pathogenesis of AD [68–72]. Similarly, many reports suggest that IGF-Ι resistance [73, 74] plays a crucial role in the pathogenesis of AD. Although serum IGF-Ι levels are increased during the early stages of disease [75], major impairments in IGF gene expression and receptor signaling have been observed in the brains of AD patients [73, 76–78]. IGFBP-3 could participate in IGF- I resistance by disrupting IGF-I-induced survival [65]. In this study, an experiment using primary murine neurons showed that IGFBP-3 precluded the IGF-induced Ser9 phosphorylation of GSK-3β (Fig. 7a and b) and that IGFBP-3 counteracted the effect of IGF-Ι to promote tau phosphorylation (Fig. 7c and d). Moreover, our study demonstrated that IGF-Ι rescued murine primary neurons from Aβ-induced cell death and that IGFBP-3 inhibited the pro-survival effect of IGF-Ι (Fig. 7g). Thus, IGFBP-3 clearly contributed to neuronal damage under our experimental conditions.

In addition, IGFBP-3 has been shown to induce apoptosis and inhibit cell growth independent of IGFs [79–81]. The apoptotic effects of IGFBP-3 have been reported in cells lacking the IGF-I receptor [80], and a function of IGFBP-3 in the nucleus has been reported [82, 83], indicating the existence of an IGF-independent mode of IGFBP-3 action.

Rensink et al. reported that IGFBP-3 levels were elevated in AD brains and that the incubation of cultured human brain pericytes with Aβ_1-40_ increased IGFBP-3 mRNA levels. High levels of IGFBP-3 have also been demonstrated to exist within hyperphosphorylated tau-positive neurons and around some Aβ plaques in AD patients [84]. In our study, increased expression of IGFBP-3 was observed in AD brains in comparison to non-AD controls (Fig. 3). Moreover, IGFBP-3 frequently existed within Aβ plaques (Fig. 3), indicating that IGFBP-3 is involved in AD pathogenesis. Accordingly, serum IGFBP-3 concentrations were increased in AD and mild cognitive impairment (MCI) patients in comparison to controls [49].

## Conclusions

In summary, our study suggested that CaN was activated by Aβ in astrocytes, leading to the release of IGFBP-3. We further demonstrated that the IGFBP-3 produced by astrocytes in turn suppresses IGF-Ι induced GSK-3β phosphorylation in neurons. This could lead tau phosphorylation in neurons. As discussed above, the molecular process that directly connects Aβ and NFT has not yet been revealed. Our study provides novel insights into the role of astrocytes in the induction of tau phosphorylation and suggests that IGFBP-3 could be an important link between Aβ and tau pathology and an important therapeutic target.

## Methods

### Antibodies and reagents

The following antibodies and reagents were used in the study:

anti-β-actin antibody, control normal mouse IgG,FK506, IGF-Ι(Sigma Aldrich, St. Louis, Missouri, USA), mouse monoclonal antibody to PHF-tau (AT8 and AT180) (Thermo Scientific, Waltham, Massachusetts, USA), and Alexa Fluor 546 goat anti-rabbit IgG conjugate and Alexa Fluor 488 goat anti-mouse IgG conjugate (life technologies, Carlsbad, California, USA). Synthetic Aβ_1–42_ peptides and Aβ_42-1_ peptides (Peptide Institute Inc., Osaka, Japan) mouse monoclonal IGFBP-3 for WB(R and D systems, Minneapolis, Minnesota, USA),rabbit polyclonal IGFBP-3 for immunostainig(Santa Cruz biotechnology, Dallas, Texas, USA), rabbit polyclonal CaN A (Enzo life science, Farmingdale, NY, USA),rabbit polyclonal AKT, p-AKT, p- GSK-3β(Ser 9) and mouse monoclonal GSK-3β(cell signaling technology, Danvers, Massachusetts, USA), FSB solution(Wako, Osaka, Japan), mouse monoclonal GFAP(Abcam, Cambridge, UK), IGFBP-3 peptide(Novus biologicals, Littleton, Colorado, USA).

### Antibody array

An antibody array (RayBio® Biotin Label-based Mouse Antibody Array I, RayBiotech, Inc., Norcross, Georgia, USA) was used according to the manufacturer’s instructions. Briefly, primary mouse astrocytes were cultured with 0.2 % fetal bovine serum (FBS) and 1 μM Aβ_1–42_ or synthetic peptide Aβ_42-1_ (inverted sequence of Aβ) for 48 h. The culture medium was collected and dialyzed with phosphate buffered saline (PBS, pH 8.0). The membranes were incubated with blocking buffer for 1 h and then with biotin-labeled medium for 2 h at room temperature. The membranes were reacted with detection buffers and exposed to X-ray films.

### Human brain sample

Human brain tissues were collected from the Tokyo Metropolitan Institute of Medical Science (Table 1). All brain samples were dissolved in radioimmune precipitation assay buffer (50 mM Tris-HCl, 150 mM NaCl, 1 % Triton X-100, 1 % Nonidet P-40, 0.5 % deoxcholate, 0.1 % SDS, pH8.0) with protease inhibitor mixture (Roche Diagnostics GmbH, Mannheim Germany) and homogenized enough on ice. These samples were then centrifuged at 14,000 × g for 20 min at 4 °C and their supernatants were collected for using Western blot analysis. The protein concentration was determined using the BCA assay.

**Table 1:** Characteristics of human brain samples

	case	age	sex	clinical diagnosis	pathological findings
non-AD	Case1	75	Male	schyzophrenia	Plaque(-),NFT stage II
	Case2	81	Female	iliopsoas abscess	Plaque(-),NFT stage II
	Case3	62	Male	schyzophrenia	Plaque(-),NFT stage II
	Case4	77	Male	liver cirrhosis	Plaque(-),NFT stageI- II
	Case5	80	Female	abdominal aortic aneurysm	Plaque(-),NFT stage II
AD	Case1	75	Alzheimer disease	NFT stageVI,C	
	Case2	68	Alzheimer disease	NFT stageVI,C	
	Case3	75	Alzheimer disease	NFT stageVI,C	
	Case4	77	Alzheimer disease	NFT stageVI,C	
	Case5	86	Alzheimer disease	NFT stageVI,C	

Clinical and histopathological information pertaining to the brain samples used in this study. We analyzed five brains from patients with neuropathology-confirmed AD and five brains from control subjects. No significant differences in age were observed between the AD and control cases. *NFT* neurofibrillary tangle

All autopsies were undertaken with informed consent and the investigation was approved by the official ethical committees of Kyoto University and of Tokyo Metropolitan Institute of Medical Science.

### Immunostaining

Human floating sections from the temporal cortex were obtained from the Tokyo Metropolitan Institute of Medical Science. After blocking in PBS containing 0.1 % Tween 20 (PBS-t) with 1 % H_2_O_2_, these sections were incubated with the primary antibodies in PBS-t for 3 days at 4 °C. These sections were washed three times (10 min each wash) with PBS and incubated with the secondary antibodies for 3 h at room temperature. Finally, the sections were washed three more times (10 min each wash). For experiments using FSB (Wako) to label amyloid plaques, the sections were then incubated with FSB for 30 min. The sections were then coverslipped with the mounting medium. To label astrocytes, mouse monoclonal anti-GFAP (1:500; Sigma-Aldrich) was used as the primary antibody and Alexa Fluor 488-conjugated goat anti-mouse (1:1000; Life Technologies) was used as the secondary antibody. To label IGFBP-3, rabbit polyclonal anti-IGFBP-3 (1:500; Santa Cruz) was used as the primary antibody and Alexa Fluor 546-conjugated mouse anti-rabbit (1:1000; Life Technologies) was used as the secondary antibody. To label CaN, goat monoclonal anti-CaN (1:500; Enzo) was used as the primary antibody and Alexa Fluor 546-conjugated goat anti-rabbit (1:1000; Life Technologies) was used as the secondary antibody. These sections were observed using a laser confocal scanning microscope (FV10i-LIV, Olympus, Tokyo, Japan).

To count the number of GFAP-positive cells that expressed CaN, we chose the following method. We tuned in to the wavelength of GFAP and took several pictures wherein some GFAP-positive cells were observed in a low-power field. We randomly selected and marked twenty cells per sample. Next, we tuned in to the wavelength of CaN and measured the ratio of the co-localization of CaN with the marked cells.

### Cell culture and cell treatment with reagent

Primary murine astrocytes were obtained from postnatal day 1 (P1) mice. Briefly, cerebral cortices were collected, rinsed 7 times with ice-cold solution minimal essential medium (SMEM) (Sigma Aldrich), dissociated by gentle trituration with fire-polished glass Pasteur pipettes, and centrifuged (1,000 rpm, 4 °C, 10 min). The cell pellet was resuspended in Dulbecco’s modified Eagle’s medium (DMEM) (Sigma Aldrich) containing 10 % fetal bovine serum (FBS) (Invitrogen, USA) and 1 % penicillin/streptomycin (PS) (Nakalai tesque, Kyoto, Japan) and filtered through a 100 μm nylon mesh. The dissociated cells were seeded into 75-cm^2^ tissue culture flasks and incubated in 5 % CO_2_ at 37 °C. The cells were fed every 3-4 days and were grown for 20-24 days until the culture became confluent. Glial cultures were shaken at 400 rpm (37 °C, 10 min). After the recovering the culture (37 °C, 2 h), the glial cultures were shaken again at a lower speed (220 rpm, 37 °C, 15 h) to dislodge microglia and oligodendrocytes. After washing 3 times with SMEM, the astrocyte cultures were detached with SMEM containing 0.25 μg/ml of trypsin (5 ml per flask, 37 °C, 15 min). An equal volume of culture medium was added to stop the trypsin digestion. The suspension was centrifuged (1,000 rpm, 24 °C, 10 min) and resuspended in DMEM with 10 % FBS and 1 % PS (DMEM/10 % FBS/1 % PS). The seeding density was 2.5 × 10^4^ cells/cm^2^. The cells present in the culture were shown to be astrocytes, with a purity of 97 ± 2 %, after characterization via immunostaining using a specific anti-GFAP primary antibody.

H4 cells (derived from human astrocytoma) were maintained in DMEM/10 % FBS/1 % PS at 37 °C in a 5 % CO_2_ incubator. mRNA was extracted from H4 cells and subjected to quantitative RT-PCR. Similarly, after treatment with 1 μM Aβ_1–42_, control peptide and 1 μM Aβ_1–42_ with 1 μM FK506 (Sigma Aldrich) for 48 h, the cell lysates and media were subjected to western blot analysis and ELISA, respectively.

Primary murine neuron cultures were obtained from the cerebral cortices of fetal mice (14-16 days of gestation) and cultured in neurobasal medium (Sigma Aldrich) supplemented with B-27 (Invitrogen) and glutamine (Sigma Aldrich). After incubation for 10 days, the cells were treated with Aβ_1–42_ (1 μM)_,_ Aβ_1–42_ (1 μM), IGF-Ι (100 ng/ml) and IGFBP-3 (100 ng/ml) for 48 h. The cells were washed twice in phosphate-buffered saline (PBS) and scraped off of the plates. The cell pellets were lysed in ice-cold TNE buffer (10 mM Tris-HCl, 150 mM NaCl, 1 % Nonidet P-40 (Roche Applied Science, Penzberg, Upper Bavaria, Germany), 1 mM EDTA, pH 7.8) containing a protease inhibitor mixture and briefly subjected to sonication. The samples were centrifuged at 14,000 × g for 5 min at 4 °C, and the supernatants were collected to obtain protein samples. The protein concentration was determined using the Bradford assay [85].

In the experiment to examine the effect of astrocyte-cultured media, we prepared two dishes, one on which primary astrocytes spread and the other on which no cell spread. The culture medium was each collected after a 48-h treatment with 1 μM Aβ. The media were centrifuged at 14,000 × g for 5 min at 4 °C, and the supernatants were collected for treatment. We treated primary neurons with the media which added 100 ng/ml IGF-Ι for 48 h and subjected the cell lysate to western blot.

### Western blotting

Protein samples were diluted with sample buffer(125 mM Tris-HCl, 4 % SDS, 2 % 2-mercaptoethanol, 20 % glycerol, 0.01 % bromophenol blue, pH6.8) and denatured at 95 °C for 3 min. Equal amounts of samples were separated by sodium dodecyl sulfate (SDS)-polyacrylamide gradient gels (5–20 %; Atto, Tokyo, Japan) in running buffer (25 mM Tris, 192 mM glycine and 0.1 % SDS), followed by Western blot, using polyvinylidene difluoride microporous membrane, blocking this membrane in *blocking One* (Nacalai tesque), and incubating with the primary antibodies in PBS containing 4 % BSA (Nacalai tesque) overnight at 4 °C. The membranes were then washed with 20 mM TBS containing 0.1 % Tween 20 (TBS-t) and incubated with horseradish peroxidase-conjugated anti-mouse or anti-rabbit IgG (GE Healthcare, Little Chalfont, UK) in TBS-t for 1 h at room temperature. The specific reaction was visualized using the ECL method (GE Healthcare).

### Real-time polymerase chain reaction (PCR) assay

Total ribonucleic acid (RNA) was extracted using ISOGEN (NIPPON GENE, Tokyo, Japan) according to the manufacturer’s protocol. cDNA was produced from each 5 μg RNA sample using a cDNA synthesis kit (GE Healthcare).

Real-time PCR primers were designed as follows: IGFBP-3: 5’-ACAGCCAGCGCTACAAAGTT-3’ and 3’-GGCTGCCCATACTTATCCAC-5’, βactin: 5’-CACACTGTGCCCATCTAC-3’ and 3’-GATCTGAAGCTCGTCCTC-5’. For the amplification of IGFBP-3 and β-actin, 5 μl of cDNA was added to the SYBR green master mix (Roche), and a real-time PCR assay was performed using a Light cycler480 (Roche).

### ELISA

The levels of IGFBP-3 in culture media were measured by human IGFBP-3 ELISA kits (R and D systems), according to the manufacturer’s instructions.

### MTT

MTT assay was performed using the MTT cell proliferation assay kit (Cayman chemical, Ann Arbor, Michigan, USA) according to the manufacturer’s instructions.

### Statistic analysis

The band densities of WB were quantified by Image J. Comparisons were performed using a Student’s *t* test. For comparison of multiparametric analysis, one-way ANOVA, followed by the post hoc analysis by Fisher’s protected least significant difference (PLSD) was used. The significance was defined by STATVIEW software. All values were given in means ± SE, and statistical significance was suggested at *p* < 0.05.

## Additional file

Additional file 1:
**The effects of IGFBP-3 on the phosphorylation of tau (AT180).** To confirm the effect of IGFBP-3 on phosphorylation of tau, the same experiment was done by using another antibody, AT180. Primary murine neurons were treated with Aβ_1–42_ (1 μM)_,_ IGF-1 (100 ng/ml) and IGFBP-3 (100 ng/ml), as designated in the figure. Western blotting analysis of primary murine neurons revealed that Aβ induced tau phosphorylation of murine primary neurons after 48 h of treatment (A and B, *n* = 4, *p* < 0.01). Phosphorylated tau normalized by β actin, is shown, converting the value of Aβ-treatment into 1(B). Although IGF-1 suppressed tau phosphorylation, IGFBP-3 counteracted the effect of IGF-1 (A and B, *p* < 0.01). * indicates statistically significant differences (**p* < 0.01). This result was compatible with the one from AT8. (PDF 222 kb)

## Abbreviations

AD: Alzheimer’s disease
Aβ: amyloid–beta
IGF: Insulin-like growth factor
IGFBP-3: Insulin-like growth factor (IGF) binding protein 3
GSK-3β: Glycogen synthase kinase-3β
APP: Amyloid precursor protein
CaN: Calcineurin
PCR: Polymerase chain reaction
RNA: Ribonucleic acid
PI3K: Phosphoinositide 3-kinase

## References

[1] Alzheimer A, Stelzmann RA, Schnitzlein HN, Murtagh FR (1995). An English translation of Alzheimer’s 1907 paper, “Uber eine eigenartige Erkankung der Hirnrinde”. Clinical anatomy (New York, NY).

[2] Tesseur I, Zou K, Esposito L, Bard F, Berber E, Can JV (2006). Deficiency in neuronal TGF-beta signaling promotes neurodegeneration and Alzheimer's pathology. J Clin Invest.

[3] Masters CL, Simms G, Weinman NA, Multhaup G, McDonald BL, Beyreuther K (1985). Amyloid plaque core protein in Alzheimer disease and Down syndrome. Proc Natl Acad Sci U S A.

[4] Mandelkow EM, Mandelkow E (1998). Tau in Alzheimer's disease. Trends Cell Biol.

[5] Crews L, Masliah E (2010). Molecular mechanisms of neurodegeneration in Alzheimer's disease. Hum Mol Genet.

[6] Beach TG, Walker R, McGeer EG (1989). Patterns of gliosis in Alzheimer's disease and aging cerebrum. Glia.

[7] Itagaki S, McGeer PL, Akiyama H, Zhu S, Selkoe D (1989). Relationship of microglia and astrocytes to amyloid deposits of Alzheimer disease. J Neuroimmunol.

[8] Glenner GG, Wong CW (1984). Alzheimer's disease and Down's syndrome: sharing of a unique cerebrovascular amyloid fibril protein. Biochem Biophys Res Commun.

[9] Glabe CC (2005). Amyloid accumulation and pathogensis of Alzheimer's disease: significance of monomeric, oligomeric and fibrillar Abeta. Subcell Biochem.

[10] Finder VH (2010). Alzheimer's disease: a general introduction and pathomechanism. J Alzheimer's Dis: JAD.

[11] Tandon A, Rogaeva E, Mullan M, St George-Hyslop PH (2000). Molecular genetics of Alzheimer's disease: the role of beta-amyloid and the presenilins. Curr Opin Neurol.

[12] Bertram L, Tanzi RE (2008). Thirty years of Alzheimer's disease genetics: the implications of systematic meta-analyses. Nat Rev Neurosci.

[13] Buee L, Bussiere T, Buee-Scherrer V, Delacourte A, Hof PR (2000). Tau protein isoforms, phosphorylation and role in neurodegenerative disorders. Brain Res Brain Res Rev.

[14] Inbar D, Belinson H, Rosenman H, Michaelson DM (2010). Possible role of tau in mediating pathological effects of apoE4 in vivo prior to and following activation of the amyloid cascade. Neurodegener Dis.

[15] Lee VM, Goedert M, Trojanowski JQ (2001). Neurodegenerative tauopathies. Annu Rev Neurosci.

[16] Mandelkow EM, Stamer K, Vogel R, Thies E, Mandelkow E (2003). Clogging of axons by tau, inhibition of axonal traffic and starvation of synapses. Neurobiol Aging.

[17] Karran E, Mercken M, De Strooper B (2011). The amyloid cascade hypothesis for Alzheimer's disease: an appraisal for the development of therapeutics. Nat Rev Drug Discov.

[18] Gamblin TC, Chen F, Zambrano A, Abraha A, Lagalwar S, Guillozet AL (2003). Caspase cleavage of tau: linking amyloid and neurofibrillary tangles in Alzheimer's disease. Proc Natl Acad Sci U S A.

[19] Garwood CJ, Pooler AM, Atherton J, Hanger DP, Noble W (2011). Astrocytes are important mediators of Abeta-induced neurotoxicity and tau phosphorylation in primary culture. Cell Death & Dis.

[20] Talantova M, Sanz-Blasco S, Zhang X, Xia P, Akhtar MW, Okamoto S (2013). Abeta induces astrocytic glutamate release, extrasynaptic NMDA receptor activation, and synaptic loss. Proc Natl Acad Sci U S A.

[21] Verkhratsky A, Olabarria M, Noristani HN, Yeh CY, Rodriguez JJ (2010). Astrocytes in Alzheimer's disease. Neurotherapeutics.

[22] Sidoryk-Wegrzynowicz M, Wegrzynowicz M, Lee E, Bowman AB, Aschner M (2011). Role of astrocytes in brain function and disease. Toxicol Pathol.

[23] Parpura V, Heneka MT, Montana V, Oliet SH, Schousboe A, Haydon PG (2012). Glial cells in (patho)physiology. J Neurochem.

[24] Nagele RG, D'Andrea MR, Lee H, Venkataraman V, Wang HY (2003). Astrocytes accumulate A beta 42 and give rise to astrocytic amyloid plaques in Alzheimer disease brains. Brain Res.

[25] Nagele RG, Wegiel J, Venkataraman V, Imaki H, Wang KC, Wegiel J (2004). Contribution of glial cells to the development of amyloid plaques in Alzheimer's disease. Neurobiol Aging.

[26] Rodriguez JJ, Olabarria M, Chvatal A, Verkhratsky A (2009). Astroglia in dementia and Alzheimer's disease. Cell Death Differ.

[27] DeWitt DA, Perry G, Cohen M, Doller C, Silver J (1998). Astrocytes regulate microglial phagocytosis of senile plaque cores of Alzheimer's disease. Exp Neurol.

[28] Schipper HM, Bennett DA, Liberman A, Bienias JL, Schneider JA, Kelly J (2006). Glial heme oxygenase-1 expression in Alzheimer disease and mild cognitive impairment. Neurobiol Aging.

[29] Kuchibhotla KV, Goldman ST, Lattarulo CR, Wu HY, Hyman BT, Bacskai BJ (2008). Abeta plaques lead to aberrant regulation of calcium homeostasis in vivo resulting in structural and functional disruption of neuronal networks. Neuron.

[30] Abramov AY, Canevari L, Duchen MR (2003). Changes in intracellular calcium and glutathione in astrocytes as the primary mechanism of amyloid neurotoxicity. J Neurosci.

[31] Abramov AY, Canevari L, Duchen MR (2004). Beta-amyloid peptides induce mitochondrial dysfunction and oxidative stress in astrocytes and death of neurons through activation of NADPH oxidase. J Neurosci.

[32] Abdul HM, Furman JL, Sama MA, Mathis DM, Norris CM (2010). NFATs and Alzheimer's Disease. Mol Cell Pharmacol.

[33] Reese LC, Taglialatela G (2011). A role for calcineurin in Alzheimer's disease. Current Neuropharmacol.

[34] Norris CM, Kadish I, Blalock EM, Chen KC, Thibault V, Porter NM (2005). Calcineurin triggers reactive/inflammatory processes in astrocytes and is upregulated in aging and Alzheimer's models. J Neurosci.

[35] Celsi F, Svedberg M, Unger C, Cotman CW, Carri MT, Ottersen OP (2007). Beta-amyloid causes downregulation of calcineurin in neurons through induction of oxidative stress. Neurobiol Dis.

[36] Abdul HM, Sama MA, Furman JL, Mathis DM, Beckett TL, Weidner AM (2009). Cognitive decline in Alzheimer's disease is associated with selective changes in calcineurin/NFAT signaling. J Neurosci.

[37] Liu F, Grundke-Iqbal I, Iqbal K, Oda Y, Tomizawa K, Gong CX (2005). Truncation and activation of calcineurin A by calpain I in Alzheimer disease brain. J Biol Chem.

[38] Wu HY, Hudry E, Hashimoto T, Kuchibhotla K, Rozkalne A, Fan Z (2010). Amyloid beta induces the morphological neurodegenerative triad of spine loss, dendritic simplification, and neuritic dystrophies through calcineurin activation. J Neurosci.

[39] Mohmmad Abdul H, Baig I, Levine H, Guttmann RP, Norris CM (2011). Proteolysis of calcineurin is increased in human hippocampus during mild cognitive impairment and is stimulated by oligomeric Abeta in primary cell culture. Aging Cell.

[40] Fernandez AM, Fernandez S, Carrero P, Garcia-Garcia M, Torres-Aleman I (2007). Calcineurin in reactive astrocytes plays a key role in the interplay between proinflammatory and anti-inflammatory signals. J Neurosci.

[41] Canellada A, Ramirez BG, Minami T, Redondo JM, Cano E (2008). Calcium/calcineurin signaling in primary cortical astrocyte cultures: Rcan1-4 and cyclooxygenase-2 as NFAT target genes. Glia.

[42] Sama MA, Mathis DM, Furman JL, Abdul HM, Artiushin IA, Kraner SD (2008). Interleukin-1beta-dependent signaling between astrocytes and neurons depends critically on astrocytic calcineurin/NFAT activity. J Biol Chem.

[43] Furman JL, Sama DM, Gant JC, Beckett TL, Murphy MP, Bachstetter AD (2012). Targeting astrocytes ameliorates neurologic changes in a mouse model of Alzheimer's disease. J Neurosci.

[44] Horsley V, Pavlath GK (2002). NFAT: ubiquitous regulator of cell differentiation and adaptation. J Cell Biol.

[45] Crabtree GR, Olson EN (2002). NFAT signaling: choreographing the social lives of cells. Cell.

[46] Akiyama H, Barger S, Barnum S, Bradt B, Bauer J, Cole GM (2000). Inflammation and Alzheimer's disease. Neurobiol Aging.

[47] Tuppo EE, Arias HR (2005). The role of inflammation in Alzheimer's disease. Int J Biochem Cell Biol.

[48] Rubio-Perez JM, Morillas-Ruiz JM (2012). A review: inflammatory process in Alzheimer's disease, role of cytokines. The Scie World J.

[49] Johansson P, Aberg D, Johansson JO, Mattsson N, Hansson O, Ahren B (2013). Serum but not cerebrospinal fluid levels of insulin-like growth factor-I (IGF-I) and IGF-binding protein-3 (IGFBP-3) are increased in Alzheimer's disease. Psychoneuroendocrinology.

[50] Rusnak F, Mertz P (2000). Calcineurin: form and function. Physiol Rev.

[51] Yu DY, Luo J, Bu F, Song GJ, Zhang LQ, Wei Q (2006). Inhibition of calcineurin by infusion of CsA causes hyperphosphorylation of tau and is accompanied by abnormal behavior in mice. Biol Chem.

[52] Fukushima T, Nakamura Y, Yamanaka D, Shibano T, Chida K, Minami S (2012). Phosphatidylinositol 3-kinase (PI3K) activity bound to insulin-like growth factor-I (IGF-I) receptor, which is continuously sustained by IGF-I stimulation, is required for IGF-I-induced cell proliferation. J Biol Chem.

[53] Hong M, Lee VM (1997). Insulin and insulin-like growth factor-1 regulate tau phosphorylation in cultured human neurons. J Biol Chem.

[54] Lesort M, Johnson GV (2000). Insulin-like growth factor-1 and insulin mediate transient site-selective increases in tau phosphorylation in primary cortical neurons. Neuroscience.

[55] Rajaram S, Baylink DJ, Mohan S (1997). Insulin-like growth factor-binding proteins in serum and other biological fluids: regulation and functions. Endocr Rev.

[56] Jones JI, Clemmons DR (1995). Insulin-like growth factors and their binding proteins: biological actions. Endocr Rev.

[57] Oh Y, Muller HL, Pham H, Rosenfeld RG (1993). Demonstration of receptors for insulin-like growth factor binding protein-3 on Hs578T human breast cancer cells. J Biol Chem.

[58] Bondy CA, Cheng CM (2004). Signaling by insulin-like growth factor 1 in brain. Eur J Pharmacol.

[59] Zhao H, Dupont J, Yakar S, Karas M, LeRoith D (2004). PTEN inhibits cell proliferation and induces apoptosis by downregulating cell surface IGF-IR expression in prostate cancer cells. Oncogene.

[60] Engelberg H (2004). Pathogenic factors in vascular dementia and Alzheimer's disease. Multiple actions of heparin that probably are beneficial. Dement Geriatr Cogn Disord.

[61] Yamada PM, Lee KW (2009). Perspectives in mammalian IGFBP-3 biology: local vs. systemic action. Am J Physiol Cell Physiol.

[62] Firth SM, Baxter RC (2002). Cellular actions of the insulin-like growth factor binding proteins. Endocr Rev.

[63] Lucas JJ, Hernandez F, Gomez-Ramos P, Moran MA, Hen R, Avila J (2001). Decreased nuclear beta-catenin, tau hyperphosphorylation and neurodegeneration in GSK-3beta conditional transgenic mice. EMBO J.

[64] Peineau S, Taghibiglou C, Bradley C, Wong TP, Liu L, Lu J (2007). LTP inhibits LTD in the hippocampus via regulation of GSK3beta. Neuron.

[65] Niikura T, Hashimoto Y, Okamoto T, Abe Y, Yasukawa T, Kawasumi M (2001). Insulin-like growth factor I (IGF-I) protects cells from apoptosis by Alzheimer's V642I mutant amyloid precursor protein through IGF-I receptor in an IGF-binding protein-sensitive manner. J Neurosci.

[66] Fernandez AM, Torres-Aleman I (2012). The many faces of insulin-like peptide signalling in the brain. Nat Rev Neurosci.

[67] Goldstein BJ (2002). Insulin resistance as the core defect in type 2 diabetes mellitus. Am J Cardiol.

[68] Jimenez S, Torres M, Vizuete M, Sanchez-Varo R, Sanchez-Mejias E, Trujillo-Estrada L (2011). Age-dependent accumulation of soluble amyloid beta (Abeta) oligomers reverses the neuroprotective effect of soluble amyloid precursor protein-alpha (sAPP(alpha)) by modulating phosphatidylinositol 3-kinase (PI3K)/Akt-GSK-3beta pathway in Alzheimer mouse model. J Biol Chem.

[69] de la Monte SM (2012). Triangulated mal-signaling in Alzheimer's disease: roles of neurotoxic ceramides, ER stress, and insulin resistance reviewed. J Alzheimers Dis.

[70] Schubert M, Gautam D, Surjo D, Ueki K, Baudler S, Schubert D (2004). Role for neuronal insulin resistance in neurodegenerative diseases. Proc Natl Acad Sci U S A.

[71] Biessels GJ, Staekenborg S, Brunner E, Brayne C, Scheltens P (2006). Risk of dementia in diabetes mellitus: a systematic review. Lancet Neurology.

[72] Tokutake T, Kasuga K, Yajima R, Sekine Y, Tezuka T, Nishizawa M (2012). Hyperphosphorylation of Tau induced by naturally secreted amyloid-beta at nanomolar concentrations is modulated by insulin-dependent Akt-GSK3beta signaling pathway. J Biol Chem.

[73] Talbot K, Wang HY, Kazi H, Han LY, Bakshi KP, Stucky A (2012). Demonstrated brain insulin resistance in Alzheimer's disease patients is associated with IGF-1 resistance, IRS-1 dysregulation, and cognitive decline. J Clin Invest.

[74] Zhang B, Tang XC, Zhang HY (2013). Alternations of central insulin-like growth factor-1 sensitivity in APP/PS1 transgenic mice and neuronal models. J Neurosci Res.

[75] Vardy ER, Rice PJ, Bowie PC, Holmes JD, Grant PJ, Hooper NM (2007). Increased circulating insulin-like growth factor-1 in late-onset Alzheimer's disease. J Alzheimers Dis.

[76] Moloney AM, Griffin RJ, Timmons S, O'Connor R, Ravid R, O'Neill C (2010). Defects in IGF-1 receptor, insulin receptor and IRS-1/2 in Alzheimer's disease indicate possible resistance to IGF-1 and insulin signalling. Neurobiol Aging.

[77] Rivera EJ, Goldin A, Fulmer N, Tavares R, Wands JR, de la Monte SM (2005). Insulin and insulin-like growth factor expression and function deteriorate with progression of Alzheimer's disease: link to brain reductions in acetylcholine. J Alzheimers Dis.

[78] Steen E, Terry BM, Rivera EJ, Cannon JL, Neely TR, Tavares R (2005). Impaired insulin and insulin-like growth factor expression and signaling mechanisms in Alzheimer's disease--is this type 3 diabetes?. J Alzheimers Dis.

[79] Ikonen M, Liu B, Hashimoto Y, Ma L, Lee KW, Niikura T (2003). Interaction between the Alzheimer's survival peptide humanin and insulin-like growth factor-binding protein 3 regulates cell survival and apoptosis. Proc Natl Acad Sci U S A.

[80] Rajah R, Valentinis B, Cohen P (1997). Insulin-like growth factor (IGF)-binding protein-3 induces apoptosis and mediates the effects of transforming growth factor-beta1 on programmed cell death through a p53- and IGF-independent mechanism. J Biol Chem.

[81] Liu B, Lee HY, Weinzimer SA, Powell DR, Clifford JL, Kurie JM (2000). Direct functional interactions between insulin-like growth factor-binding protein-3 and retinoid X receptor-alpha regulate transcriptional signaling and apoptosis. J Biol Chem.

[82] Li W, Fawcett J, Widmer HR, Fielder PJ, Rabkin R, Keller GA (1997). Nuclear transport of insulin-like growth factor-I and insulin-like growth factor binding protein-3 in opossum kidney cells. Endocrinology.

[83] Jaques G, Noll K, Wegmann B, Witten S, Kogan E, Radulescu RT (1997). Nuclear localization of insulin-like growth factor binding protein 3 in a lung cancer cell line. Endocrinology.

[84] Rensink AA, Gellekink H, Otte-Holler I, ten Donkelaar HJ, de Waal RM, Verbeek MM (2002). Expression of the cytokine leukemia inhibitory factor and pro-apoptotic insulin-like growth factor binding protein-3 in Alzheimer's disease. Acta Neuropathol.

[85] Bradford MM (1976). A rapid and sensitive method for the quantitation of microgram quantities of protein utilizing the principle of protein-dye binding. Anal Biochem.

