# Supplementary figures and images for: The participation of insulin-like growth factor-binding protein 3 released by astrocytes in the pathology of Alzheimer’s disease
Source: Mol Brain. 2015 Dec 4;8:82. doi: 10.1186/s13041-015-0174-2 (PMC4670528; doi:10.1186/s13041-015-0174-2)

Figure S

p-tau  
(AT180)

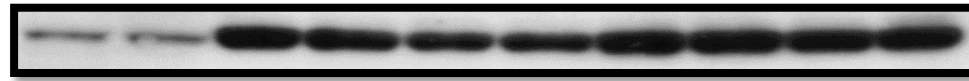

β actin

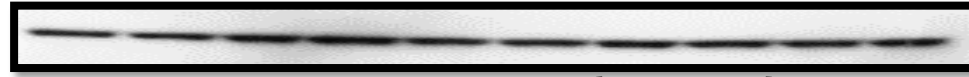

Aβ (1μM)

IGF-1 (100ng/ml)

IGFBP-3 (100ng/ml)

|   |   |   |   |   |
|---|---|---|---|---|
| - | + | + | + | + |
| - | - | + | + | - |
| - | - | - | + | + |

A

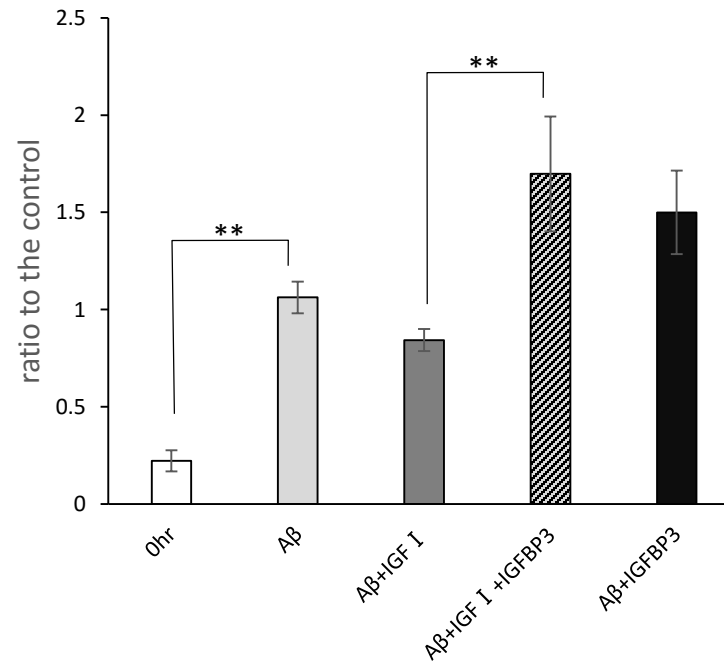

B phosphorylation of tau (AT180)

Supplement: Additional file 1: — The effects of IGFBP-3 on the phosphorylation of tau (AT180). To confirm the effect of IGFBP-3 on phosphorylation of tau, the same experiment was done by using another antibody, AT180. Primary murine neurons were treated with Aβ1–42 (1 μM), IGF-1 (100 ng/ml) and IGFBP-3 (100 ng/ml), as designated in the figure. Western blotting analysis of primary murine neurons revealed that Aβ induced tau phosphorylation of murine primary neurons after 48 h of treatment (A and B, n = 4, p < 0.01). Phosphorylated tau normalized by β actin, is shown, converting the value of Aβ-treatment into 1(B). Although IGF-1 suppressed tau phosphorylation, IGFBP-3 counteracted the effect of IGF-1 (A and B, p < 0.01). * indicates statistically significant differences (*p < 0.01). This result was compatible with the one from AT8. (PDF 222 kb) [file 13041_2015_174_MOESM1_ESM.pdf]
